# Strengthening primary health care to achieve universal health coverage

**DOI:** 10.1016/j.lanepe.2024.100897

**Published:** 2024-04-04

**Authors:** The Lancet Regional Health – Europe

Health systems worldwide are grappling with a critical challenge: the pursuit of universal health coverage (UHC), wherein all individuals can access a comprehensive range of quality health services without experiencing financial hardship. Globally, 4.5 billion people lack full coverage of essential health services. A robust primary health care system is essential to realising UHC. Encompassing health promotion, disease prevention, treatment, rehabilitation, and palliative care, primary health care serves as the cornerstone of a sustainable and resilient health system. It is estimated that, through a primary health care approach, 90% of essential interventions for UHC can be delivered. However, even in the wealthiest countries in Europe, millions struggle with out-of-pocket payments for primary health care, resulting in impoverishment for 1%–12% of households and catastrophic health spending ranging from 1% to 20%.

Investing in primary health care is crucial for achieving UHC. However, in a recent disappointing development, it was announced at the European Council summit in Brussels that €1 billion would be cut from the €5.3 billion budget for the EU4Health programme. This reduction not only constitutes a 20% decrease in the overall health budget for the European Union, but also undermines the priorities and strategic programming of health financing for the remaining years of the Framework until 2027.

Amidst budget constraints, unlocking the full potential of primary health care to achieve UHC hinges on adeptly implementing effective and cost-efficient strategies. Building a resilient primary health care workforce, nurturing a people-centred health system, and ensuring digital transformation are pivotal for strengthening primary health care.

The health-care workforce forms the foundation of any health system, yet Europe is currently facing a workforce crisis with a shortage of about 1.8 million health-care workers, a number that could surge to 4 million by 2030. This shortage is particularly acute in primary health care as the number of people leaving or retiring from the workforce is higher than that of new people joining. In England alone, nearly 5 million patients wait over 2 weeks for a general practitioner (GP) appointment monthly, while one in three GPs plan to quit within the next few years.

Implementing key measures outlined in the WHO Framework for Action 2023–2030, particularly within the Retain and Recruit action, is vital for strengthening the primary health-care workforce. Improving working conditions, such as reducing heavy workloads, limiting excessive hours, offering contract flexibility, and ensuring fair remuneration, is essential. These actions not only promote the mental health and wellbeing of health-care workers, but also increase job appeal. Supportive policies, such as integration support for migrant health-care workers, incentives for local recruitment, expanding primary health care curriculum in medical education to promote interest, and providing skills training for migrants can further enhance primary health care careers’ attractiveness.

Considering the challenges of an ageing population and increasing burden of chronic diseases, transitioning from current fragmented, disease-focused care to integrated, comprehensive care can address multifaceted health needs effectively. Therefore, nurturing a people-centred health system that integrates services across different sectors to achieve the highest attainable level of health, is key to strengthening primary health care. For example, in Kazakhstan, the adoption of people-centred care in primary health care involving multidisciplinary teams of GPs, nurses, social workers, and psychologists, led to a 46% decrease in non-communicable diseases related mortality, surpassing the average of 31% in WHO European member states. This success underscores the feasibility and benefits of a people-centred primary health care approach, which can eventfully lead to achievement of UHC by ensuring comprehensive care and reducing treatment costs.

While digital transformation holds promise for enhancing health-care access and quality, integrating it into existing primary health care systems poses substantial challenges, primarily due to resistance to change. In Greece, a paperless ePrescription system was adopted in 2012, which initially faced resistance but was accelerated by the COVID-19 pandemic, highlighting its potential to revolutionise primary care delivery. Overcoming such resistance is essential for countries that have not yet embraced the digital revolution underway worldwide, since digitalisation can advance UHC by improving efficiency and access to care and ensuring high-quality service delivery.

Europe is falling short in its pursuit of UHC, as evidenced by the rise in catastrophic health spending between 2000 and 2019, which affected 74 million people. Without a concerted focus on strengthening primary health care, the ambitious goal of achieving UHC by 2030 could slip further from reach. It is imperative to advocate for a paradigm shift that elevates primary health care to the forefront of health-care agendas.

